# Circulating Tumor DNA as a Marker for Treatment Response in Metastatic Melanoma Patients Using Next-Generation Sequencing—A Prospective Feasibility Study

**DOI:** 10.3390/cancers13123101

**Published:** 2021-06-21

**Authors:** Marina Berger, Andrea Thueringer, Doritt Franz, Nadia Dandachi, Emina Talakić, Georg Richtig, Erika Richtig, Peter Michael Rohrer, Lukas Koch, Ingrid Hildegard Wolf, Catharina Koch, Barbara Margaretha Rainer, Maximilian Koeller, Martin Pichler, Hanno Gerritsmann, Karl Kashofer, Ariane Aigelsreiter

**Affiliations:** 1 Department of Dermatology, Medical University of Graz, 8036 Graz, Austria; marina.berger2@kages.at (M.B.); erika.richtig@medunigraz.at (E.R.); peter.rohrer@medunigraz.at (P.M.R.); lukas.koch@medunigraz.at (L.K.); ingrid.wolf@medunigraz.at (I.H.W.); catharina.koch@medunigraz.at (C.K.); barbara.rainer@medunigraz.at (B.M.R.); 2 Diagnostic and Research Institute of Pathology, Medical University of Graz, 8010 Graz, Austria; andrea.thueringer@medunigraz.at (A.T.); doritt.franz@medunigraz.at (D.F.); 3 Department of Internal Medicine, Division of Oncology, Medical University of Graz, 8036 Graz, Austria; nadia.dandachi@medunigraz.at (N.D.); georg.richtig@medunigraz.at (G.R.); martin.pichler@medunigraz.at (M.P.); 4 Department of Radiology, Medical University of Graz, 8036 Graz, Austria; emina.talakic@medunigraz.at; 5 Department of Pathology, Medical University of Vienna, 1090 Vienna, Austria; maximilian.koeller@meduniwien.ac.at; 6 Medical Affairs Oncology, Novartis Pharma GmbH, 1020 Vienna, Austria; hanno.gerritsmann@novartis.com

**Keywords:** metastatic melanoma, circulating tumor DNA, next-generation sequencing, custom panels, treatment monitoring

## Abstract

**Simple Summary:**

Despite the improvement of the prognosis of metastatic melanoma patients through the implementation of targeted and immunotherapies, there is a need to identify biomarkers to predict and monitor treatment response. Therefore, we performed sequencing of paired melanoma tissue biopsies and circulating tumor DNA (ctDNA) from 149 plasma samples using two custom next-generation sequencing (NGS) AmpliSeq™ HD panels to determine the level of concordance. We aimed to evaluate whether ctDNA analysis with NGS could predict and monitor treatment response in a cohort of metastatic melanoma patients; NGS allowed for a comprehensive analysis of cancer-associated mutations in serial plasma samples with high sensitivity. Although a trend could be seen that mutant allele frequency levels over time correlated with or even preceded radiological response to treatment, this finding was not statistically significant in our cohort. Our study demonstrates that NGS gene panels might be useful for treatment monitoring with ctDNA in melanoma patients.

**Abstract:**

We prospectively performed a longitudinal analysis of circulating tumor DNA (ctDNA) from 149 plasma samples and CT scans in Stage III and IV metastatic melanoma patients (*n* = 20) treated with targeted agents or immunotherapy using two custom next-generation sequencing (NGS) Ion AmpliSeq™ HD panels including 60 and 81 amplicons in 18 genes, respectively. Concordance of matching cancer-associated mutations in tissue and plasma was 73.3%. Mutant allele frequency (MAF) levels showed a range from 0.04% to 28.7%, well detectable with NGS technologies utilizing single molecule tagging like the AmpliSeq™ HD workflow. Median followup time of the tissue and/or plasma positive cohort (*n* = 15) was 24.6 months and median progression-free survival (PFS) was 7.8 months. Higher MAF ≥ 1% at baseline was not significantly associated with a risk of progression (Odds Ratio = 0.15; *p* = 0.155). Although a trend could be seen, MAF levels did not differ significantly over time between patients with and without a PFS event (*p* = 0.745). Depending on the cell-free DNA amount, NGS achieved a sensitivity down to 0.1% MAF and allowed for parallel analysis of multiple mutations and previously unknown mutations. Our study indicates that NGS gene panels could be useful for monitoring disease burden during therapy with ctDNA in melanoma patients.

## 1. Introduction

Despite the advances in drug developments within the last years, metastatic melanoma still has a poor prognosis. Nevertheless, the progression-free survival (PFS) and overall survival (OS) of patients with metastatic melanoma improved considerably with the introduction of targeted therapies and immunotherapies [1,2,3,4]. Despite the impressive activity of these novel treatment approaches, not all patients respond or have a durable response [5,6,7]. Therefore, there is a paramount interest to identify effective biomarkers to monitor disease course and ultimately personalize therapy.

Large scale sequencing brought to light the complex mutational landscape of melanoma and identified frequent mutations in BRAF, NRAS, TP53, CDKN2A, KIT, NF1, and SF3B1 genes [8,9]. Melanoma is a genetically heterogeneous tumor and shows a complex evolutionary pattern from initiation by ultraviolet light exposure to progression to metastatic disease [10]. In addition, the clonal evolution of melanoma may continue during systemic therapy leading to the activation of resistance mechanisms and subsequent relapse [11,12]. 

Circulating tumor DNA (ctDNA) appears to be a promising noninvasive, repeatable, and systemic biomarker for predicting treatment response and monitoring throughout therapy in metastatic melanoma. The prognostic utility of ctDNA was demonstrated in metastatic melanoma patients with undetectable or low ctDNA at baseline, and throughout therapy, it was associated with better response and longer PFS in patients treated with targeted therapies or immunotherapies [13,14]. These data are mainly based on real-time quantitative polymerase chain reaction (qPCR) assays and droplet digital PCR (ddPCR), while data using next-generation sequencing (NGS) gene panels for monitoring melanoma patients via ctDNA during therapy are sparse. To date, only two studies employed a targeted custom melanoma NGS panel to analyze matching tumor-associated mutations in tissue and ctDNA in melanoma [15,16]. Diefenbach et al. [15] used a targeted custom panel (123 covering 30 genes) in a cohort of 74 stage III and IV melanoma patients with detection of ctDNA in 84% of stage IV and 47% of stage III patients, with a limit of detection for mutant allele frequency (MAF) of 0.2%. The first study to employ a custom melanoma NGS panel (950 amplicons covering 30 genes) from a cohort (*n* = 24) of stage IV melanoma patients found a confirmed driver mutation in 70% of matching plasma samples [16]. AmpliSeq™ HD is a proprietary system to perform single molecular barcoding in the Ion Torrent™ workflow by Thermo Fisher Scientific Inc. Waltham, MA, USA [17]. It introduces molecular barcodes on both ends of DNA fragments generated in very few primer annealing and extension steps. After purification of these extension fragments, the NGS library is amplified using universal A&P primers and sequenced on Ion Torrent™ sequencers. During basecalling, the molecular barcodes are removed from the sequence and saved as tags with each individual read in the resulting BAM file. Variant calling is a twostep process, first normal variant calling is performed on all reads and in a second step the validity of each variant is assessed taking the individual tags on the supporting molecules into account. Only variants present in all the reads of a read family originating from a single molecule are true positives and should therefore be counted towards the final allele frequency.

The aim of this prospective feasibility study was to develop and test the performance of targeted custom melanoma NGS AmpliSeq™ HD panels in a cohort of metastatic melanoma patients. Secondly, we aimed to evaluate whether ctDNA analysis with NGS could predict and monitor radiological treatment response. To further determine the level of concordance of tissue and liquid biopsy, we performed sequencing of paired melanoma tissue biopsies and ctDNA from plasma using two custom NGS AmpliSeq™ HD panels (Table S1). Since studies already showed the existence of tumor heterogeneity [18,19,20] as well as the occurrence of acquired resistance mutations under ongoing therapy [12,21,22,23], we included patients without melanoma-associated mutations detectable in the tissue biopsy in our study as a separate cohort.

## 2. Materials and Methods

### 2.1. Patients

Metastatic melanoma patients (*n* = 31) treated with either BRAF/MEK-targeted therapy or immunotherapy were enrolled in this prospective study between September 2017 and November 2019 at the University Clinic for Dermatology and Venerology of the Medical University of Graz, Austria. Patients were treated with either dabrafenib/trametinib or pembrolizumab, nivolumab, ipilimumab monotherapy, or a combination of ipilimumab/nivolumab in currently approved doses. Dosing regimens were administered according to U.S. Food and Drug Administration (FDA) approved doses of targeted therapy or immunotherapy (PD-1 and CTLA-4 antibodies). Patients included were not treated with targeted therapy or immunotherapy two years before study inclusion.

### 2.2. Tissue Samples

Tissue biopsy mutation profiles were obtained prior to study inclusion as well as plasma sampling and identified using custom AmpliSeq™ panels covering the main mutational hotspots in cutaneous and uveal melanoma (for details, see Table S1).

### 2.3. Plasma Sample Preparation and Circulating Cell Free DNA(cfDNA) Extractions

Blood samples were collected into cfDNA BCT^®^ (Streck, La Vista, NE, USA) tubes and stored at room temperature. Plasma was separated within 72 h by centrifugation at 500 g for 20 min, followed by a second centrifugation at 2500 g for 20 min, and then stored at −80 °C until extraction. CtDNA was isolated from 1–5 mL of plasma using the QIAamp Circulating Nucleic Acid Kit (Qiagen, Hilden, Germany) according to the manufacturer’s instructions. CtDNA was eluted in 50 μL nuclease free water and was subsequently quantified using the Quant-it PicoGreen dsDNA Reagents (Life Technologies, Carlsbad, CA, USA), according to the manufacturer’s instructions and stored at −80 °C. As allele frequencies of mutations were expected to be low in ctDNA samples, a custom melanoma panel was designed using the AmpliSeq™ HD technology. The panel initially consisted only of targeting mutations common in cutaneous melanoma (HD-Melanom v3) but was subsequently expanded to also include mutations present in uveal melanoma (HD-Melanom v1—for details, see Table S1). NGS libraries were prepared from up to 30 ng ctDNA using the AmpliSeq™ HD library kit (CatNr: A37694, Thermo Fisher Scientific Inc. Waltham, MA, USA) according to the manufacturer’s instructions. Libraries were quantified using the Ion Library TaqMan Quantitation Kit (CatNr: 4468802, Thermo Fisher Scientific Inc., Waltham, MA, USA) and sequenced on Ion S5XL using the 200 bp workflow to a depth leading to approximately 20,000 coverage of each amplicon in the panel (~3.5 Mio. reads per sample). NGS data were analyzed by Torrent variant caller; variants were annotated by Annovar [24]. As some false positive reads and mispriming artefacts remain in the raw variant caller results, particularly below a threshold of 1–3% MAF, we thus rigorously manually reviewed the results of the variant caller and removed known PCR artifacts—identifiable because they are present in all samples—as well as very low read calls which did not match concomitant analysis of the tumor tissue. Thus, we are confident that we only report real true positive variants in our analysis.

### 2.4. Routine Laboratory Parameter Determination

Serum tubes to analyze routine laboratory parameters such as S100, lactate dehydrogenase (LDH), and C-reactive protein (CRP) were also collected at every patient visit. The level of S100 was determined by using an electrochemiluminescence immunoassay “ECLIA”. The level of CRP was determined by a turbidimetric assay and the LDH level was analyzed by photometry.

### 2.5. Disease Characteristics and Response Assessment

Patient demographics and clinicopathologic features including S100 and LDH levels at baseline (date of first liquid biopsy) and throughout therapy, mutation status in the tissue biopsy, ECOG performance status, and AJCC tumor stage at baseline were collected. Investigator-determined objective response was assessed radiologically at two to three monthly intervals with computed tomography (CT) scans alone or where indicated, with magnetic resonance imaging (MRI) of the brain using RECIST 1.1 criteria [25], and classified as having a complete response (CR), partial response (PR), stable disease (SD), or progressive disease (PD). Patients who did not have restaging imaging due to clinical disease progression were classified as progressive disease (PD) and included in the analysis.

### 2.6. Statistical Analysis

Statistical analyses were performed using Stata (Mac version 16.1, Stata Corp., Houston, TX, USA). Continuous variables were summarized as medians (interquartile range (IQR)), and categorical variables were reported as absolute counts and percentages. MAF levels were considered as a continuous variable or were dichotomized into a binary variable (detectable versus not detectable or <1% versus ≥1%). The association between MAF levels at baseline and clinical benefit was assessed with Wilcoxon’s rank sum tests (continuous variables) and *x*^2^, or with Fisher’s exact tests (categorical variables). The association between clinical benefit and MAF was analyzed using univariate logistic regression. Median followup time was estimated with the reverse Kaplan–Meier estimator [26]. Probabilities of PFS and OS were computed using Kaplan–Meier estimators and compared between two or more groups using log-rank tests. Due to the low number of patients and events, multivariate analysis was not performed. A linear mixed-effects regression model with a random-intercept at the patient level was performed to examine changes in blood biomarkers during progression on treatment. The model parameters were estimated using maximum likelihood, and an independent variance-covariance structure was assumed for the random effects. To analyze the correlation between MAF levels and S100, LDH, and CRP, Spearman’s rank-based correlation coefficient was used.

## 3. Results

### 3.1. Cohort and Sample Characteristics

**Figure 1:**
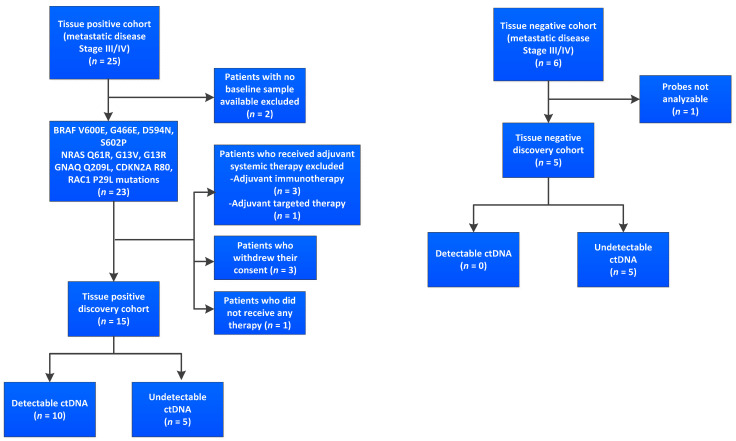
Flowchart showing total number of patients recruited for study and reasons for exclusion from further evaluation and ctDNA analysis. The type of mutations in tissue positive cohort are at baseline (pretreatment). Tissue positive cohort included patients with at least one melanoma-associated mutation in tissue specimen. Tissue negative cohort consisted of patients without a mutation in tissue specimen. ctDNA: circulating tumor DNA.

In total, 165 plasma samples were collected from 31 metastatic melanoma patients, including a baseline sample for 29 patients, collected 0–28 days prior to therapy initiation. Tissue mutation profiling of all patients recruited for the study revealed the presence of BRAF V600E, BRAF G466E, BRAF D594N, BRAF S602P, NRAS Q61R, NRAS Q61L, NRAS G13V, NRAS G13R, GNAQ Q209L, CDKN2A R80, and RAC1 P29L mutations. Longitudinal plasma samples were obtained for 23/31 (74.2%) patients, collected over several time points up to nine weeks from treatment commencement. Eleven of 31 patients were excluded from further evaluation (as illustrated in Figure 1): Two patients did not have a baseline sample available; in four patients, the liquid biopsy sample was taken after surgical resection and they received adjuvant therapy; three patients withdrew their consent; in one patient, the samples could not be analyzed due to technical issues, and one patient did not receive the planned immunotherapy, but received radiation inste.

Of the finally evaluable 20 melanoma patients (as illustrated in Table 1), two (10%) had stage III and 18 (90%) had stage IV melanoma. The median age of the cohort was 70 years (IQR 67–76), and most patients were male (*n* = 13; 65%). Fifteen patients had cutaneous melanoma, while the other five had uveal melanoma (*n* = 1), mucosal melanoma (*n* = 1), and melanoma of unknown primary (*n* = 3). Clinically detectable lymph node metastases were present in 1/2 (50%) stage III patients, whereas 1/2 (50%) patients had in-transit cutaneous and subcutaneous metastases only. Of the patients with stage IV disease, one patient had M1a, eight had M1b, seven had M1c, and two had M1d disease (with concurrent extracranial metastases in 2/2 patients). At baseline, S100 was elevated in 9/20 (45%) and LDH was elevated in 10/20 (50%) patients. 5/20 (25%) patients were treated with targeted therapies (dabrafenib/trametinib in combination) and 15/20 (75%) received immunotherapies (pembrolizumab, nivolumab, ipilimumab, or nivolumab/ipilimumab in combination).

### 3.2. Evaluation of the Performance of the Custom Melanoma Panels

**Figure 2:**
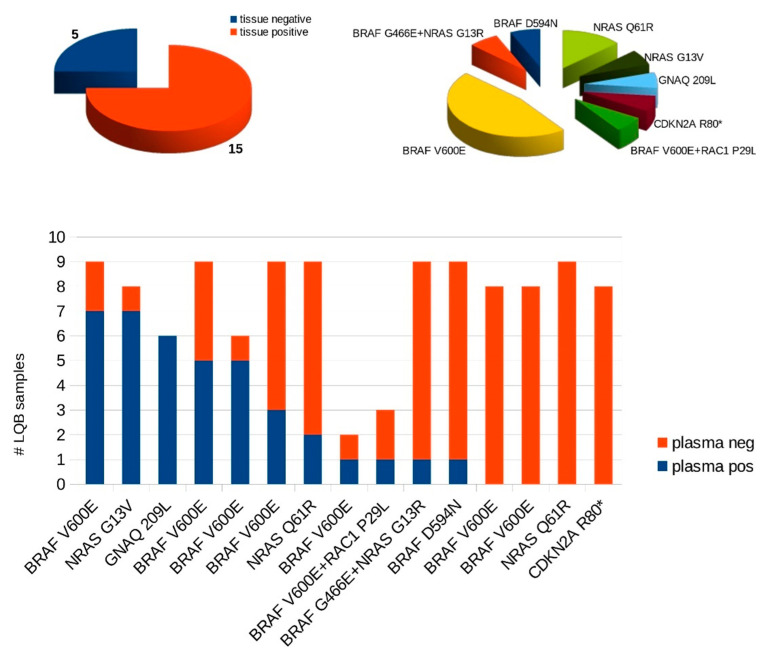
Pie charts showing ratio of tissue positive to tissue negative patients in our cohort (*n* = 20), as well as proportions of detected cancer-associated mutations in tissue specimens. Bar chart illustrates number of ctDNA positive or negative plasma samples per patient in tissue positive patient cohort (*n* = 15). Each bar chart represents one patient. #LQB, cumulative number of liquid biopsy samples; *, stop mutation.

Longitudinal analysis of serial ctDNA (149 blood samples) through NGS was able to track BRAF mutations in 6/20 (30%), NRAS mutations in 2/20 (10%), and GNAQ mutations in 1/20 (5%) patients, as well as BRAF/RAC1 (1/20; 5%) and BRAF/NRAS (1/20; 5%) double mutations. Therefore, in 15/20 (75%) patients, at least one mutation was found in the tissue specimen (tissue positive cohort), and at least one matching ctDNA mutation (MAF > 0%) could be detected at baseline in 11 (73.3%) of these patients, as displayed in Figure 2.

Analysis of ctDNA revealed a previously unknown GNAQ Q209L driver mutation in a tissue negative patient (ID17) with unknown primary. As the GNAQ mutation hotspot was not included in the originally used tissue melanoma panel (Core Cancer Panel V10, Table S1) developed for cutaneous melanoma, the tissue was therefore reanalyzed using the Ion Torrent™ AmpliSeq™ Panel for cutaneous and uveal melanoma (Melanom as illustrated in Panel 4, Table S1). Retesting of the patient’s tissue revealed the GNAQ mutation already preexisted in the tissue before treatment commencement. The remaining five tissue and plasma negative patients (tissue negative cohort) were excluded from the following analyses.

**Figure 3:**
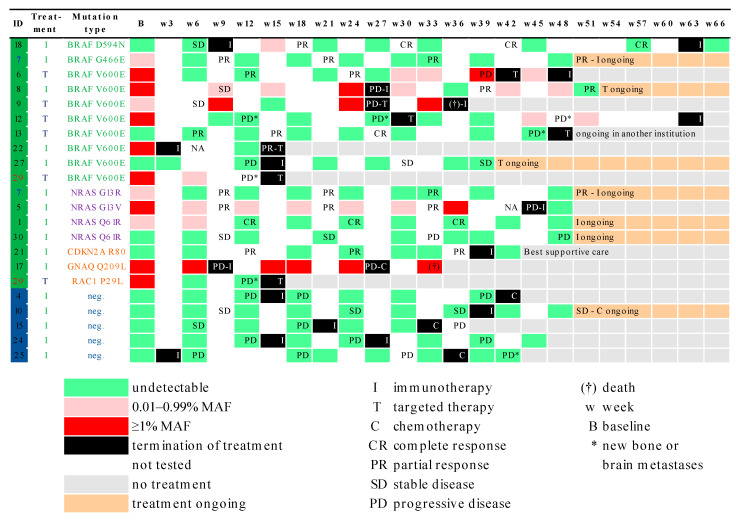
Heatmap showing mutation status and longitudinal ctDNA followup of individual patients at different time points during treatment (immunotherapy or targeted therapy) grouped by mutational status in the tissue/plasma (at least one melanoma-associated mutation detectable in tissue/plasma or tissue/plasma negative). Each row is an individual patient, and each column is a time point during followup (every three weeks). Termination of each treatment type is marked in black boxes. Grey boxes signify that patient did not receive treatment with either immunotherapy, targeted therapy, or chemotherapy anymore. MAF levels are marked by green boxes (undetectable), pink boxes (0.01–0.99% MAF), and red boxes (≥1% MAF). Time points at which there was no evaluation of ctDNA are shown in white color. Two patients had two different mutations in tissue as well as in the plasma samples and are listed twice in heatmap (ID 7 in blue and ID 29 in red). MAF: mutant allele frequency.

Overall, mean MAF levels were 7.7% (range 0.04% to 28.7%, as illustrated in Table S2) including two double mutations and in 18 (20%) samples MAF was ≥1% (as illustrated in Figure 3). NGS achieved a sensitivity down to 0.1% MAF used in routine diagnostic setting and lower for research purposes in this project if the cfDNA amount in the plasma sample was sufficient to analyze at least 3000 molecules.

### 3.3. Correlation of MAF Levels with Radiological Response

**Figure 4:**
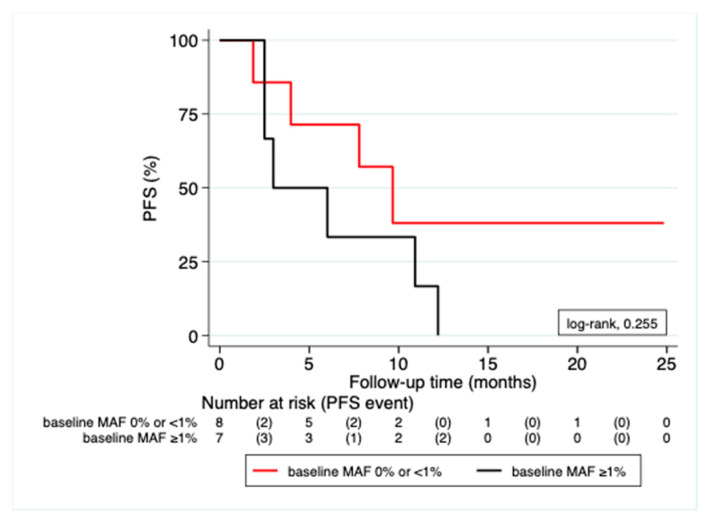
Comparison between groups of patients with high (≥1%) versus low/undetectable (<1%) baseline MAF for PFS. Kaplan–Meier curve for PFS according to MAF levels at baseline (pretreatment)—high versus low/undetectable. PFS: progression-free survival; MAF: mutant allele frequency.

Median followup duration for the cohort was 24.6 months (95% CI; 16.6–25.1); nine (56.3%) patients were alive at time of analysis, and five (31.3%) patients had ongoing treatment response. Median PFS was 7.8 months (95% CI; 2.5–12.2). CtDNA analyses of baseline and on-therapy samples revealed two distinct patient profiles. The first group (*n* = 8) consisted of patients with undetectable or low MAF levels (<1%) at baseline; the second group (*n* = 7) had a high MAF (≥1%) at baseline. Clinical characteristics across the two patient groups were similar for age, sex, tumor stage, prevalence of brain metastases, mutational distribution, and treatment type (as illustrated in Table 1). No significant association could be found between higher MAF (≥1%) at baseline and risk of progression (OR = 0.150; 95% CI 0.56–8.06; *p* = 0.155) in our cohort. Interestingly, a weak association (*p* = 0.084) between detectable ctDNA at baseline and site of metastasis in the lung was observed. Baseline MAF levels were not associated with PFS (HR = 2.06; 95% CI 0.57–7.51; *p* = 0.269) as shown in Figure 4.

**Figure 5:**
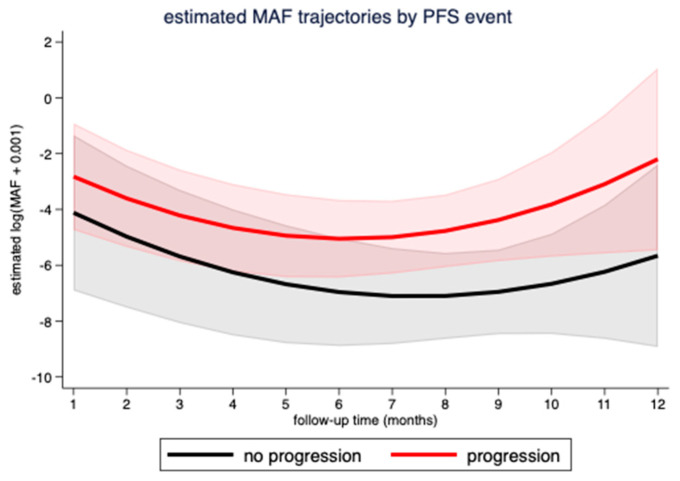
Evolution of MAF levels over followup time in patients with or without progression under treatment. Data are from a mixed model using 74 MAF measurements from 15 patients. MAF: mutant allele frequency; PFS: progression-free survival.

To investigate the longitudinal evolution of MAF levels under treatment, we used a mixed model with quadratic growth of MAF values, a random intercept at the patient level, and a random slope for linear followup time. We studied 74 MAF measurements from 15 patients from baseline until disease progression or censoring alive without a PFS event (average number of MAF measurements per patient: 5, range 1–9). According to this model, the estimated MAF levels were higher for patients with a PFS event at any follow-up time compared to patients without a PFS event (estimated difference = 2.21, 95%CI: 0.19–4.24, *p* = 0.032). As shown in Figure 5, MAF levels significantly changed over followup time (joint effect of linear and quadratic followup time *p*-value < 0.001). In detail, MAF levels initially decreased over time but started to increase later. However, although a trend could be seen (as shown in Figure 5), MAF trajectories did not differ significantly over time between patients with and without a PFS event (overall interaction *p*-value for linear and quadratic followup time = 0.745).

**Figure 6:**
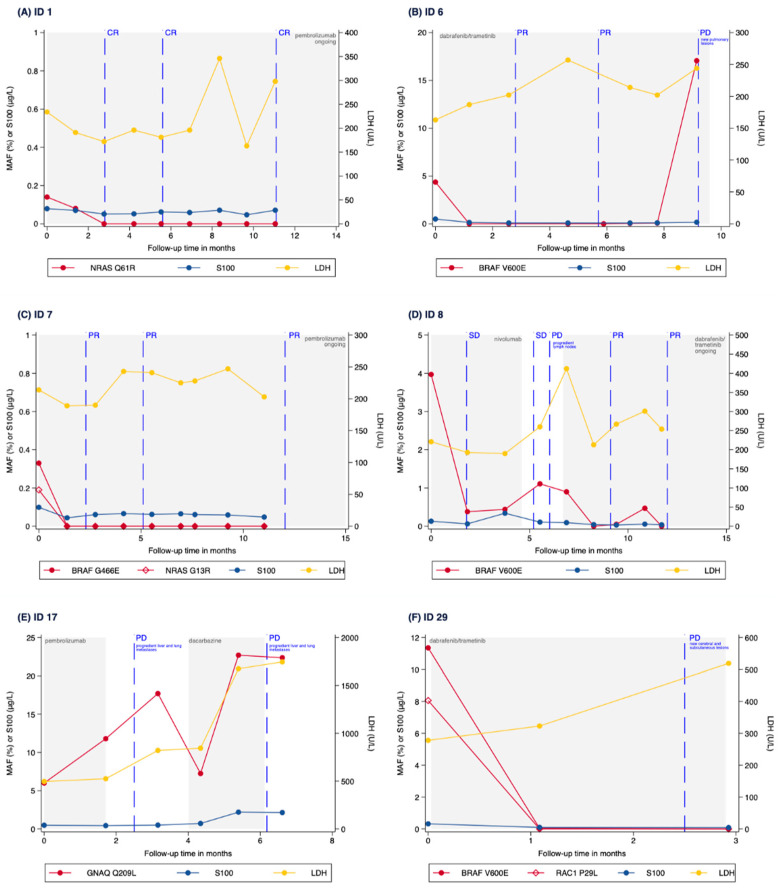
(**A**) Time course of a patient (ID 1) with MAF levels monitored through a NRAS Q61R mutation during pembrolizumab treatment. MAF decreases under pembrolizumab treatment and becomes undetectable, while radiologically displaying complete response to treatment. (**B**) Time course of a patient (ID 6) with MAF levels monitored through a BRAF V600E mutation in plasma during dabrafenib/trametinib treatment. MAF decreased and became undetectable prior to radiological evidence of partial response to treatment. In the further course, MAF increased again, while CT scans showed newly developed lesions in the lung. (**C**) Time course of a patient (ID 7) with MAF levels monitored through both a BRAF G466E and a NRAS G13R mutation in plasma during pembrolizumab treatment. MAFs of both mutations fell and became undetectable after initiation of pembrolizumab, preceding CT scans displaying partial response to treatment. (**D**) Time course of a patient (ID 8) with MAF levels monitored through a BRAF V600E mutation in plasma during nivolumab and subsequent dabrafenib/trametinib treatment as well as during a treatment pause in between. The MAF of the BRAF mutation rose before radiological disease progression and fell after change of treatment regimen. (**E**) Time course of a patient (ID 17) with uveal melanoma and MAF levels monitored through a GNAQ Q209L mutation in plasma during pembrolizumab and subsequent dacarbazine (DTIC) treatment as well as during a treatment pause in between. MAF of the mutation increased during and after pembrolizumab treatment and showed a decline after initiation of dacarbazine chemotherapy. However, soon after this, the patient displayed again increasing GNAQ Q209L MAF as well as increasing S100 and LDH levels, preceding the radiological progression under chemotherapy. (**F**) Time course of a patient (ID 29) with MAF levels monitored through both a BRAF V600E and a RAC1 P29L mutation in plasma during dabrafenib/trametinib treatment. MAFs of both mutations fell during the first weeks of treatment and remained undetectable in further course, while CT scans showed new subcutaneous and brain lesions. Scales on axes are not normalized to better display dynamics. MAF: mutant allele frequency; LDH: lactate dehydrogenase; CR: complete response; PR: partial response; SD: stable disease; PD: progressive disease.

Time courses of 6 patients are presented in Figure 6, showing the MAF, S100, and LDH levels as well as the radiological responses during therapy with different treatment types. In most cases shown, MAF levels correlated well with the response and even preceded radiological disease progression. Interestingly, this finding could be observed regardless of which therapy the patients received (targeted therapy, immunotherapy, or chemotherapy as second- or third-line treatment). In patients that initially or durably responded to treatment, MAF levels went down or became undetectable during the first weeks of therapy (ID1, ID6, ID7), while MAF levels increased prior to radiological disease progression (ID6, ID7, ID8, ID17), as can be discerned from Figure 6.

In two patients with a double mutation in the tissue specimen, both mutations could be identified in the plasma. CtDNA analysis revealed a BRAF/NRAS mutation in patient ID7 and a BRAF/RAC1 mutation in patient ID29. Longitudinal analysis of ctDNA showed a similar course of both mutations during therapy (shown in Figure 6). Of note, in two patients with predominantly soft tissue and brain metastases, ctDNA was not detectable in plasma. CtDNA analysis of patient ID12 with a BRAF mutation showed a high MAF level (≥1%) at baseline with no longer detectable MAF and radiologically completely regressed lung and lymph node metastases, but new bone lesions developed during targeted therapy. The same patient continued to have very low MAF levels during subsequent immunotherapy, despite radiological evidence of new brain metastases. Furthermore, patient ID29 with a BRAF/RAC1 double mutation had high MAF levels at baseline, which were no longer detectable during targeted therapy, while the patient had radiologically regressed lung metastases, but newly developed brain lesions (as illustrated in Figure 6).

**Figure 7:**
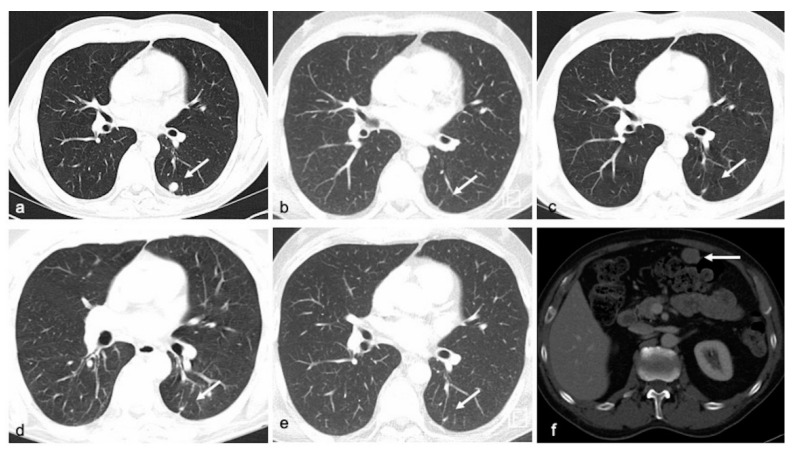
CT scans showing the patient’s (ID 6) response to treatment with dabrafenib/trametinib (**a**) Axial CT images of lung demonstrate a secondary lesion (target lesion) in left lower lobe in baseline examination. Patient also has known osseous metastases (nontarget lesions). (**b**–**d**) 3, 6, and 9 months after therapy, there is still no evidence of the pulmonary nodule, the osseous metastases remain unchanged, in summary corresponding to partial response. (**e**,**f**) 13 months after therapy, there is still no evidence of the pulmonary nodule, but a new mesenteric implant was found, suggestive of progressive disease, which later was confirmed to represent metastatic tissue.

To assess radiological response towards therapeutic interventions, CT scans and, where indicated, MRI of the brain were used (as illustrated in Figure 7) and response was classified according to RECIST 1.1 criteria.

### 3.4. Correlation of MAF Levels with S100, LDH and CRP Levels

MAF levels correlated with S100 levels (r = 0.361; *p* < 0.001, *N* = 143) as well as with CRP levels (r = 0.304; *p* < 0.001, *N* = 144). Interestingly, MAF levels did not correlate with LDH levels (r = 0.030; *p* = 0.726, *N* = 144) in our cohort.

## 4. Discussion

In the last 10 years, there was a significant change in the treatment of metastatic melanoma with the introduction of targeted therapies and immunotherapies. Although these therapies show high response rates [4,5,6], there is still a need for reliable biomarkers to predict and monitor treatment response. One possible approach is the use of ctDNA to obtain patient-specific genomic information that can be monitored in real-time during treatment.

The use of PCR-based assays, such as ddPCR, provides high analytical sensitivity in detecting ctDNA and the ability to longitudinally monitor driver mutations. But typically, only a limited number of mutations can be detected in parallel, and the alterations have to be previously known. In contrast, custom NGS panels can analyze multiple mutations in parallel, allowing for the discovery of other cancer-associated mutations coexisting in the tumor or metastases (due to molecular heterogeneity) or newly acquired mutations (e.g., resistance mutations during treatment). The development of targeted NGS cancer gene panels optimized for the detection of ctDNA provides both the flexibility of multiple mutation analysis coupled with a sensitivity that approaches or even matches ddPCR [27].

From this perspective, sequencing of ctDNA via NGS-based technologies will hopefully become a reliable tool in melanoma that already succeeded in identifying alterations at a frequency as low as one mutant copy in several thousand wild-type copies in other tumor entities [28,29]. Although there are some studies using NGS in melanoma, very few studies [15,16] tested the performance of custom Ion AmpliSeq™ HD panels or their clinical utility in metastatic melanoma patients to date.

In our study using two custom Ion AmpliSeq™ HD panels, NGS was able to track melanoma driver mutations during extended treatment periods in a cohort of metastatic melanoma patients with high sensitivity. Ion Torrent™ AmpliSeq™ HD allowed to reliably analyze a large number of relevant mutation hotspots in liquid biopsy samples collected in a real-world clinical setting in our cohort. Sensitivity of the analysis was sufficient to track tumor mutations in most of the patients. CtDNA was detected in 73.3% of our patients combined, a detection rate equivalent to other melanoma studies [15,21,30]. Importantly, a trend could be seen that MAF changes correlated with treatment events (as illustrated in Figure 5) and even preceded the radiological response in several cases (as illustrated in Figure 6). The fact that higher MAF (≥1%) at baseline was not significantly associated with neither risk of progression nor with worse PFS may be explained by the small number of patients analyzed.

The increasing focus on tumor heterogeneity showed the importance of assessing the full spectrum of molecular alterations in different disease sites to enable appropriate treatment decisions. However, in patients with advanced disease, tissue samples from multiple sites are generally not feasible. In contrast, plasma may overcome these limitations and represent a summation of molecular changes across multiple disease sites [21]. However, in our cohort, no acquired mutations were detected during treatment in any of the patients. We hypothesize that number of genes in a panel might play an important role in this as responsible mutations for newly developed subclones in metastases could be missed if the panel is too small. Eventually, more novel mutations could be found during therapy if the panels were expanded. This still must be evaluated in further research.

In patient ID 17, a GNAQ mutation was found in the plasma with no mutation previously identified in the tissue specimen. The location of the primary tumor was unknown in this patient, but the discovery of the GNAQ mutation indicated that the patient may have a uveal melanoma. Although in this case the GNAQ mutation could be identified in the tissue at retesting with another panel including uveal melanoma mutations, we suggest that liquid biopsy could also help with identifying the primary tumor in patients with unknown primary and negative tissue sampling. Nevertheless, further studies are needed to confirm this presumption. In patients with soft tissue and brain metastases, ctDNA was not a reliable biomarker, as levels of ctDNA remained low or undetectable even with a substantial increase in tumor burden. These findings are consistent with other studies that revealed low or undetectable ctDNA levels despite extensive cerebral and/or soft tissue metastases [14,21,31,32,33]. The underrepresentation of subcutaneous and cerebral disease sites in the plasma poses a significant limitation to ctDNA monitoring, and recognition of this limitation is essential if ctDNA monitoring is to be integrated into routine clinical practice. Thus, we suggest a combination of S100 and LDH levels as well as CT scans, and where indicated, MRI or FDG-PET scans, to monitor radiologic and metabolic disease burden with ctDNA through NGS to track molecular evolution and provide complementary information of disease burden in advanced melanoma patients.

Perez–Guijarro et al. [34] characterized several different mouse models of melanoma. In their study they list BRAF, NRAS, ERBB4, TP53, NF1, and PTEN as well as GNAQ and GNA11 as frequently mutated genes according to TCGA. Our Ampliseq HD panel covers the majority of these genes, but also includes other genes important for classification and treatment of melanoma. Marie et al. [35] presented an insightful transcriptome analysis implicating several important pathways in the evolution of melanoma. However, transcriptome analysis is not possible from liquid biopsies; therefore, our panel design focuses on frequent, clinically relevant DNA mutation hotspots and utilizes a molecular barcoding approach, achieving very high sensitivity, which is imperative for reliable and successful analysis of ccfDNA.

Our Ampliseq panel uses molecular barcoding which allows for reliably deduplicating reads and count real single molecules during analysis. Using these numbers, amplification of genes in tumor tissue DNA could be detected. We paid close attention to molecule counts in our analyses of the plasma samples in this study to possibly gain insight into copy number changes (CNV) but could not detect convincing amplification signals in any of the samples.

The aim of this feasibility study was to develop and test the performance of targeted custom melanoma NGS AmpliSeq™ HD panels in a cohort of metastatic melanoma patients. In our study, NGS using custom Ion AmpliSeq™ HD panels allowed for a comprehensive analysis of cancer-associated mutations in serial plasma. Depending on the cfDNA amount, NGS achieved a sensitivity down to 0.1% MAF and allowed for parallel analysis of multiple mutations and previously unknown mutations. Secondly, we aimed to evaluate whether ctDNA analysis with NGS could predict and monitor radiological treatment response. Although a trend could be seen that MAF levels over time correlated with or even preceded radiological response to treatment, this finding was not statistically significant in our cohort. Interestingly, MAF levels correlated with S100 and CRP but not with LDH in our study.

## 5. Conclusions

Our study indicates that NGS gene panels might be valuable tools for monitoring treatment response during targeted and immunotherapy with ctDNA in metastatic melanoma patients. Larger prospective studies are needed to validate our findings concerning the custom Ion AmpliSeq™ HD panels.

## Figures and Tables

**Table 1:** Baseline patient characteristics of cohort (*n* = 20).

Subcategories	Baseline ctDNA Low * MAF < 1% (*n* = 8)	Baseline ctDNA High MAF ≥ 1% (*n* = 7)	Tissue/Plasma Neg. (*n* = 5)	Total (*n* = 20)
Age	70 (67–78)	70 (59–76)	70 (68–75)	71 (67–76)
Gender				
Male	5 (62.5)	5 (71.4)	3 (60)	13 (65)
Female	3 (37.5)	2 (28.6)	2 (40)	7 (35)
ECOG				
0	7 (87.5)	3 (42.8)	4 (80)	14 (70)
1	1 (12.5)	2 (28.6)	0 (0)	3 (15)
2	0 (0)	1 (14.3)	0 (0)	1 (5)
Unknown	0 (0)	1 (14.3)	1 (20)	2 (10)
Melanoma Type				
Cutaneous melanoma	8 (100)	4 (57.1)	3 (60)	15 (75)
Uveal melanoma	0 (0)	0 (0)	1 (10)	1 (5)
Mucosal melanoma	0 (0)	1 (14.3)	0 (0)	1 (5)
Unknown primary	0 (0)	2 (28.6)	1 (20)	3 (15)
Tissue localization				
Primary	4 (50)	4 (57.1)	2 (40)	10 (50)
Metastasis	4 (50)	3 (42.9)	2 (40)	9 (45)
Missing	0 (0)	0 (0)	1 (20)	1 (5)
Tissue mutation				
BRAF	5 (62.5)	5 (71.4)	0 (0)	10 (50)
NRAS	2 (25)	1(14.3)	0 (0)	3 (15)
CDKN2A	1 (12.5)	0 (0)	0 (0)	1 (5)
GNAQ	0 (0)	1 (14.3)	0 (0)	1 (5)
neg.	0 (0)	0 (0)	5 (100)	5 (25)
AJCC tumor stage				
IIIC/IIID	1 (12.5)	1 (14.3)	0 (0)	2 (10)
IV	7 (87.5)	6 (85.7)	5 (100)	18 (90)
pT				
T0	1 (12.5)	0 (0)	0 (0)	1 (5)
pT1	0 (0)	1 (14.3)	0 (0)	1 (5)
pT2	3 (37.5)	2 (28.6)	0 (0)	5 (25)
pT3	1 (12.5)	1 (14.3)	2 (40)	4 (20)
pT4	3 (37.5)	1 (14.3)	1 (20)	5 (25)
pTX/Missing	0 (0)	2 (28.6)	2 (40)	4 (20)
pN				
N0	4 (50)	2 (28.6)	1 (20)	7 (35)
N1	1 (12.5)	2 (28.6)	3 (60)	6 (30)
N2	3 (37.5)	0 (0)	1 (20)	4 (20)
N3	0 (0)	3 (42.8)	0 (0)	3 (15)
M stage				
M0	1 (12.5)	1 (14.3)	0 (0)	2 (10)
M1a	0 (0)	1 (14.3)	0 (0)	1 (5)
M1b	5 (62.5)	1 (14.3)	2 (40)	8 (40)
M1c	2 (25)	2 (28.6)	3 (60)	7 (35)
M1d	0 (0)	2 (28.6)	0 (0)	2 (10)
Number of metastatic sites				
1	3 (37.5)	2 (28.6)	2 (40)	7 (35)
2	4 (50)	3 (42.8)	1 (20)	8 (40)
≥3	1 (12.5)	2 (28.6)	2 (40)	5 (25)
S100				
≤1× ULN	8 (100)	0 (0)	3 (60)	11 (55)
>1× ULN	0 (0)	7 (100)	2 (40)	9 (45)
LDH				
≤1× ULN	7 (87.5)	3 (42.9)	0 (0)	10 (50)
>1× ULN	1 (12.5)	4 (57.1)	5 (100)	10 (50)
Treatment type				
Targeted therapy	2 (25)	3 (42.9)	0 (0)	5 (25)
Immunotherapy	6 (75)	4 (57.1)	5 (100)	15 (75)

Data are presented as median (IQR) for continuous variables as age and absolute frequencies (%) for categorical variables. IQR, interquartile range; MAF, mutant allele frequency; ULN, upper limit of normal; LDH, lactate dehydrogenase. * Includes 5 patients with undetectable ctDNA (MAF = 0%) at baseline.

## Data Availability

No new data were created or analyzed in this study. Data sharing is not applicable to this article.

## Supplementary Materials

The following are available online at https://www.mdpi.com/article/10.3390/cancers13123101/s1, Table S1: description of the panels used for tissue (primary tumor or metastasis) and for plasma (cfDNA) mutation profiling in our study cohort; Table S2: list of MAF as well as S100, LDH, and CRP levels.

## References

[1] Larkin J., Ascierto P.A., Dréno B., Atkinson V., Liszkay G., Maio M., Mandalà M., Demidov L., Stroyakovskiy D., Thomas L. (2014). Combined vemurafenib and cobimetinib in BRAF-mutated melanoma. N. Engl. J. Med..

[2] Flaherty K.T., Infante J.R., Daud A., Gonzalez R., Kefford R.F., Sosman J., Hamid O., Schuchter L., Cebon J., Ibrahim N. (2012). Combined BRAF and MEK inhibition in melanoma with BRAF V600 mutations. N. Engl. J. Med..

[3] Hodi F.S., O’Day S.J., McDermott D.F., Weber R.W., Sosman J.A., Haanen J.B., Gonzalez R., Robert C., Schadendorf D., Hassel J.C. (2010). Improved survival with ipilimumab in patients with metastatic melanoma. N. Engl. J. Med..

[4] Ribas A., Puzanov I., Dummer R., Schadendorf D., Hamid O., Robert C., Hodi F.S., Schachter J., Pavlick A.C., Lewis K.D. (2015). Pembrolizumab versus investigator-choice chemotherapy for ipilimumab-refractory melanoma (KEYNOTE-002): A randomised, controlled, phase 2 trial. Lancet Oncol..

[5] Schreuer M., Jansen Y., Planken S., Chevolet I., Seremet T., Kruse V., Neyns B. (2017). Combination of dabrafenib plus trametinib for BRAF and MEK inhibitor pretreated patients with advanced BRAFV600-mutant melanoma: An open-label, single arm, dual-centre, phase 2 clinical trial. Lancet Oncol..

[6] Uhrig M., Hassel J.C., Schlemmer H.-P., Ganten M.-K. (2013). Therapy response assessment in metastatic melanoma patients treated with a braf inhibitor. Adapted choi criteria can reflect early therapy response better than does RECIST. Acad. Radiol..

[7] Hodi F.S., Hwu W.-J., Kefford R., Weber J.S., Daud A., Hamid O., Patnaik A., Ribas A., Robert C., Gangadhar T.C. (2016). Evaluation of immune-related response criteria and RECIST v1.1 in patients with advanced melanoma treated with pembrolizumab. J. Clin. Oncol..

[8] The Cancer Genome Atlas Network (2015). Genomic Classification of Cutaneous Melanoma The Cancer Genome Atlas Network HHS Public Access. Cell.

[9] Hayward N., Wilmott J., Waddell N., Johansson P.A., Field M.A., Nones K., Patch A.-M., Kakavand H., Alexandrov L.B., Burke H. (2017). Whole-genome landscapes of major melanoma subtypes. Nat. Cell Biol..

[10] Shain A.H., Yeh I., Kovalyshyn I., Sriharan A., Talevich E., Gagnon A., Dummer R., North J.P., Pincus L.B., Ruben B.S. (2015). The genetic evolution of melanoma from precursor lesions. N. Engl. J. Med..

[11] Rizos H., Menzies A.M., Pupo G.M., Carlino M.S., Fung C., Hyman J., Haydu L.E., Mijatov B., Becker T.M., Boyd S.C. (2014). BRAF inhibitor resistance mechanisms in metastatic melanoma: Spectrum and clinical impact. Clin. Cancer Res..

[12] Shi H., Hugo W., Kong X., Hong A., Koya R.C., Moriceau G., Chodon T., Guo R., Johnson D.B., Dahlman K.B. (2014). Acquired resistance and clonal evolution in melanoma during BRAF inhibitor therapy. Cancer Discov..

[13] Santiago-Walker A., Gagnon R., Mazumdar J., Casey M., Long G.V., Schadendorf D., Flaherty K.T., Kefford R., Hauschild A., Hwu P. (2016). Correlation of BRAF mutation status in circulating-free DNA and tumor and association with clinical outcome across four BRAFi and MEKi clinical trials. Clin. Cancer Res..

[14] Lee J., Long G.V., Boyd S., Lo S., Menzies A.M., Tembe V., Guminski A., Jakrot V., Scolyer R.A., Mann G. (2017). Circulating tumour DNA predicts response to anti-PD1 antibodies in metastatic melanoma. Ann. Oncol..

[15] Diefenbach R., Lee J., Menzies A., Carlino M., Long G., Saw R., Howle J., Spillane A., Scolyer R., Kefford R. (2020). Design and testing of a custom melanoma next generation sequencing panel for analysis of circulating tumor DNA. Cancers.

[16] Calapre L., Giardina T., Robinson C., Reid A.L., Al-Ogaili Z., Pereira M.R., McEvoy A., Warburton L., Hayward N., Khattak M.A. (2019). Locus-specific concordance of genomic alterations between tissue and plasma circulating tumor DNA in metastatic melanoma. Mol. Oncol..

[17] Ion AmpliSeq HD Technology for Targeted Sequencing. https://www.thermofisher.com/at/en/home/products-and-services/promotions/life-science/ampliseq-hd.html.

[18] Diefenbach R.J., Lee J.H., Strbenac D., Yang J.Y.H., Menzies A.M., Carlino M.S., Long G.V., Spillane A.J., Stretch J.R., Saw R.P.M. (2019). Analysis of the whole-exome sequencing of tumor and circulating tumor DNA in metastatic melanoma. Cancers.

[19] Bakhoum S.F., Cantley L.C. (2018). The Multifaceted role of chromosomal instability in cancer and its microenvironment. Cell.

[20] Prasetyanti P.R., Medema J.P. (2017). Intra-tumor heterogeneity from a cancer stem cell perspective. Mol. Cancer.

[21] Gray E.S., Rizos H., Reid A.L., Boyd S.C., Pereira M.R., Lo J., Tembe V., Freeman J., Lee J., Scolyer R.A. (2015). Circulating tumor DNA to monitor treatment response and detect acquired resistance in patients with metastatic melanoma. Oncotarget.

[22] Zaretsky J.M., Garcia-Diaz A., Shin D.S., Escuin-Ordinas H., Hugo W., Hu-Lieskovan S., Torrejon D.Y., Abril-Rodriguez G., Sandoval S., Barthly L. (2016). Mutations associated with acquired resistance to PD-1 blockade in melanoma. N. Engl. J. Med..

[23] Czarnecka A.M., Bartnik E., Fiedorowicz M., Rutkowski P. (2020). Targeted therapy in melanoma and mechanisms of resistance. Int. J. Mol. Sci..

[24] Wang K., Li M., Hakonarson H. (2010). ANNOVAR: Functional annotation of genetic variants from high-throughput sequencing data. Nucleic Acids Res..

[25] Eisenhauer E.A., Therasse P., Bogaerts J., Schwartz L.H., Sargent D., Ford R., Dancey J., Arbuck S., Gwyther S., Mooney M. (2009). New response evaluation criteria in solid tumours: Revised RECIST guideline (version 1.1). Eur. J. Cancer.

[26] Schemper M., Smith T.L. (1996). A note on quantifying follow-up in studies of failure time. Control. Clin. Trials.

[27] Diefenbach R.J., Lee J.H., Rizos H. (2019). Monitoring melanoma using circulating free DNA. Am. J. Clin. Dermatol..

[28] Forshew T., Murtaza M., Parkinson C., Gale D., Tsui D.W.Y., Kaper F., Dawson S.-J., Piskorz A.M., Jimenez-Linan M., Bentley D. (2012). Noninvasive identification and monitoring of cancer mutations by targeted deep sequencing of plasma DNA. Sci. Transl. Med..

[29] Kinde I., Wu J., Papadopoulos N., Kinzler K.W., Vogelstein B. (2011). Detection and quantification of rare mutations with massively parallel sequencing. Proc. Natl. Acad. Sci. USA.

[30] Girotti M.R., Gremel G., Lee R., Galvani E., Rothwell D., Viros A., Mandal A.K., Lim K.H.J., Saturno G., Furney S.J. (2016). Application of sequencing, liquid biopsies, and patient-derived xenografts for personalized medicine in melanoma. Cancer Discov..

[31] Bettegowda C., Sausen M., Leary R.J., Kinde I., Wang Y., Agrawal N., Bartlett B., Wang H., Luber B., Alani R.M. (2014). Detection of circulating tumor DNA in early- and late-stage human malignancies. Sci. Transl. Med..

[32] De Mattos-Arruda L., Mayor R., Ng C.K.Y., Weigelt B., Martínez-Ricarte F., Torrejon D., Oliveira M., Arias A., Raventos C., Tang J. (2015). Cerebrospinal fluid-derived circulating tumour DNA better represents the genomic alterations of brain tumours than plasma. Nat. Commun..

[33] Wong S.Q., Raleigh J.M., Callahan J., Vergara I.A., Ftouni S., Hatzimihalis A., Colebatch A.J., Li J., Semple T., Doig K. (2017). Circulating tumor DNA analysis and functional imaging provide complementary approaches for comprehensive disease monitoring in metastatic melanoma. JCO Precis. Oncol..

[34] Pérez-Guijarro E., Yang H.H., Araya R.E., El Meskini R., Michael H.T., Vodnala S.K., Marie K.L., Smith C., Chin S., Lam K. (2020). Multimodel preclinical platform predicts clinical response of melanoma to immunotherapy. Nat. Med..

[35] Marie K.L., Sassano A., Yang H.H., Michalowski A.M., Michael H.T., Guo T., Tsai Y.C., Weissman A.M., Lee M.P., Jenkins L.M. (2020). Melanoblast transcriptome analysis reveals pathways promoting melanoma metastasis. Nat. Commun..

