# Supplementary material for: Circulating Tumor DNA as a Marker for Treatment Response in Metastatic Melanoma Patients Using Next-Generation Sequencing—A Prospective Feasibility Study
Source: Cancers (Basel). 2021 Jun 21;13(12):3101. doi: 10.3390/cancers13123101 (PMC8233754; doi:10.3390/cancers13123101)
Supplement: Supplementary file 1 [file cancers-13-03101-s001.zip › Supplementary Table S2.pdf]

ID	Therapy	Mutation 1	Mutation 2	No.	Maf 1	Maf 2	Response	S100 (-0,105µg/l)	LDH (-240 U/L)	CRP (-5mg/L)
**1**	I	NRAS Q61R		1	0,14		NA	0,079	234	0,6
**1**	I	NRAS Q61R		2	0,08		NA	0,070	191	**25,9**
**1**	I	NRAS Q61R		3	0		CR	0,051	172	1,1
**1**	I	NRAS Q61R		4	0		CR	0,052	196	0,6
**1**	I	NRAS Q61R		5	0		CR	0,062	181	1,2
**1**	I	NRAS Q61R		6	0		CR	0,059	196	0,6
**1**	I	NRAS Q61R		7	0		CR	0,071	**346**	1,0
**1**	I	NRAS Q61R		8	0		CR	0,047	163	0,9
**1**	I	NRAS Q61R		9	0		**CR**	0,071	**298**	1,4
**4**	I	neg.		1	0		NA	0,060	**714**	**74,0**
**4**	I	neg.		2	0		NA	0,070	**953**	**74,3**
**4**	I	neg.		3	0		**PD**	0,088	**1150**	**9,1**
**4**	C	neg.		4	0		PD	0,096	**1724**	**56,2**
**4**	C	neg.		5	0		PD	**0,107**	**2180**	**81,3**
**4**	C	neg.		6	0		PD	**0,164**	**3140**	**22,1**
**4**	C	neg.		7	0		PD	**0,199**	**5438**	**80,8**
**5**	I	NRAS G13V		1	26,03		NA	**0,376**	**272**	2,8
**5**	I	NRAS G13V		2	0,05		PR	0,105	239	1,5
**5**	I	NRAS G13V		3	0,72		PR	0,102	**248**	2,2
**5**	I	NRAS G13V		4	0,54		PR	**0,108**	208	**10,8**
**5**	I	NRAS G13V		5	0,16		PR	**0,130**	222	2,0
**5**	I	NRAS G13V		6	0,16		PR	**0,109**	231	3,5
**5**	I	NRAS G13V		7	1,08		PR	**0,187**	212	4,8
**5**	I	NRAS G13V		8	NA		PR	**0,134**	173	1,9
**5**	N	NRAS G13V		9	0		**PD**	**0,120**	**317**	**144,6**
**6**	T	BRAF V600E		1	4,36		NA	**0,496**	163	**79,5**
**6**	T	BRAF V600E		2	0		NA	**0,143**	187	4,2
**6**	T	BRAF V600E		3	0		PR	0,087	202	**20,1**
**6**	T	BRAF V600E		4	0		PR	0,087	**257**	4,5
**6**	T	BRAF V600E		5	0		PR	NA	NA	NA
**6**	T	BRAF V600E		6	0,04		PR	0,080	214	**7,5**
**6**	T	BRAF V600E		7	0,07		PR	0,098	202	**5,7**
**6**	T	BRAF V600E		8	17,06		**PD**	**0,161**	**244**	2,9
**6**	I	BRAF V600E		9	0,41		PD	**0,133**	201	**16,1**
**7**	I	BRAF G466E	NRAS G13R	1	0,33	0,19	NA	0,099	214	**6,1**
**7**	I	BRAF G466E	NRAS G13R	2	0	0	NA	0,044	189	2,8
**7**	I	BRAF G466E	NRAS G13R	3	0	0	PR	0,061	190	3,7
**7**	I	BRAF G466E	NRAS G13R	4	0	0	PR	0,066	**243**	3,1
**7**	I	BRAF G466E	NRAS G13R	5	0	0	PR	0,062	**241**	**6,0**
**7**	I	BRAF G466E	NRAS G13R	6	0	0	PR	0,065	225	**15,8**
**7**	I	BRAF G466E	NRAS G13R	7	0	0	PR	0,061	228	**9,1**
**7**	I	BRAF G466E	NRAS G13R	8	0	0	PR	0,059	**247**	**16,8**
**7**	I	BRAF G466E	NRAS G13R	9	0	0	**PR**	0,048	203	**18,8**
**8**	I	BRAF V600E		1	3,97		NA	**0,131**	221	**18,9**
**8**	I	BRAF V600E		2	0,38		SD	0,062	193	**29,1**
**8**	I	BRAF V600E		3	0,44		**SD**	**0,340**	190	**8,0**
**8**	N	BRAF V600E		4	1,11		PD	**0,107**	**260**	**24,5**
**8**	T	BRAF V600E		5	0,9		NA	0,094	**412**	**41,6**
**8**	T	BRAF V600E		6	0		NA	0,042	213	**9,7**
**8**	T	BRAF V600E		7	0,05		PR	0,032	**267**	4,5
**8**	T	BRAF V600E		8	0,47		PR	0,055	**301**	**6,8**
**8**	T	BRAF V600E		9	0		PR	0,037	**254**	3,5
**9**	T	BRAF V600E		1	0,83		NA	**0,131**	221	**18,9**
**9**	T	BRAF V600E		2	1,37		SD	0,062	193	**29,1**
**9**	T	BRAF V600E		3	0		**SD**	**0,340**	190	**8,0**
**9**	N	BRAF V600E		4	2,44		SD	**0,107**	**260**	**24,5**
**9**	I	BRAF V600E		5	19,3		PD	0,094	**412**	**41,6**
**10**	I	neg.		1	0		NA	0,053	**334**	**10,8**
**10**	I	neg.		2	0		NA	0,098	**316**	**6,3**
**10**	I	neg.		3	0		SD	0,074	**362**	**9,5**
**10**	I	neg.		4	0		SD	NA	NA	NA
**10**	I	neg.		5	0		SD	0,076	**311**	**6,8**
**10**	I	neg.		6	0		SD	0,068	**301**	**5,8**
**10**	I	neg.		7	0		**SD**	0,079	**411**	**9,8**
**10**	C	neg.		8	0		SD	0,074	**389**	**10,1**
**10**	C	neg.		9	0		SD	0,082	**980**	3,8
**12**	T	BRAF V600E		1	2,91		NA	**0,119**	175	**96,2**
**12**	T	BRAF V600E		2	0		NA	NA	NA	NA
**12**	T	BRAF V600E		3	0		**PD***	**NA**	215	4,4
**12**	I	BRAF V600E		4	0		PD	0,032	251	4,6
**12**	I	BRAF V600E		5	0		PD*	0,028	155	**2,0**
**12**	I	BRAF V600E		6	0		PD	0,040	215	4,6

ID	Therapy	Mutation 1	Mutation 2	No.	Maf 1	Maf 2	Response	S100 (-0,105µg/l)	LDH (-240 U/L)	CRP (-5mg/L)
**12**	I	BRAF V600E		7	0		PD	0,048	**279**	2,3
**12**	I	BRAF V600E		8	0,24		PD*	0,039	215	4,5
**12**	I	BRAF V600E		9	0,25		PD	0,035	**429**	**5,3**
**13**	T	BRAF V600E		1	0		NA	0,099	205	1,3
**13**	T	BRAF V600E		2	0		PR	**0,117**	**293**	0,8
**13**	T	BRAF V600E		3	0		PR	**0,120**	**471**	0,6
**13**	T	BRAF V600E		4	0		PR	0,099	**365**	2,4
**13**	T	BRAF V600E		5	0		PR	0,101	**296**	2,2
**13**	T	BRAF V600E		6	0		CR	0,094	**288**	2,3
**13**	T	BRAF V600E		7	0		NA	0,088	**304**	1,3
**13**	T	BRAF V600E		8	0		**PD***	0,088	**322**	2,5
**15**	I	neg.		1	0		NA	**0,112**	**466**	3,1
**15**	I	neg.		2	0		SD	**1,640**	**275**	**5,5**
**15**	I	neg.		3	0		**SD**	**0,458**	**295**	**56,2**
**15**	N	neg.		4	0		PD	**1,340**	207	**91,0**
**15**	C	neg.		5	0		PD	**3,930**	**361**	**226,3**
**15**	C	neg.		6	0		PD	**3,570**	**512**	**72,7**
**17**	I	QNAQ Q209L		1	6		NA	**0,484**	**496**	4,6
**17**	I	QNAQ Q209L		2	11,8		**PD**	**0,433**	**525**	3,8
**17**	N	QNAQ Q209L		3	17,7		PD	**0,499**	**821**	**11,3**
**17**	C	QNAQ Q209L		4	7,25		PD	**0,716**	**845**	**21,8**
**17**	C	QNAQ Q209L		5	22,69		PD	**2,190**	**1674**	**43,2**
**17**	C	QNAQ Q209L		6	22,36		PD	**2,140**	**1745**	**59,1**
**18**	I	BRAF D594N		1	0		**NA**	0,062	**266**	2,7
**18**	I	BRAF D594N		2	0		SD	0,063	**384**	3,0
**18**	N	BRAF D594N		3	0		SD	0,068	**371**	**79,6**
**18**	N	BRAF D594N		4	0		PR	0,065	228	2,4
**18**	N	BRAF D594N		5	0		PR	0,040	222	**7,1**
**18**	N	BRAF D594N		6	0		CR	0,039	229	**6,8**
**18**	N	BRAF D594N		7	0		CR	0,038	223	**6,0**
**18**	N	BRAF D594N		8	0		CR	0,046	203	3,5
**18**	N	BRAF D594N		9	0		NA	0,049	**251**	1,8
**21**	I	CDKN2A R80		1	0		NA	0,068	201	1,5
**21**	I	CDKN2A R80		2	0		NA	0,056	192	**5,2**
**21**	I	CDKN2A R80		3	0		PR	0,063	197	4,8
**21**	I	CDKN2A R80		4	0		PR	0,062	211	1,4
**21**	I	CDKN2A R80		5	0		PR	62,000	181	1,2
**21**	I	CDKN2A R80		6	0		PR	0,044	200	1,4
**21**	I	CDKN2A R80		7	0		**PR**	0,039	187	2,9
**21**	N	CDKN2A R80		8	0		PR	0,049	147	1,6
**22**	I	BRAF V600E		1	28,69		**NA**	**5,380**	**827**	**76,9**
**22**	T	BRAF V600E		2	NA		NA	**0,160**	**361**	**10,3**
**22**	T	BRAF V600E		3	0		PR	0,105	205	**7,4**
**24**	I	neg.		1	0		NA	0,024	**247**	0,9
**24**	I	neg.		2	0		**NA**	0,033	**243**	**243,0**
**24**	N	neg.		3	0		PD	**0,112**	**429**	0,6
**24**	I	neg.		4	0		PD	0,039	**360**	1,5
**24**	I	neg.		5	0		PD	0,044	**269**	0,6
**24**	N	neg.		6	0		PD	0,026	**290**	1,0
**24**	N	neg.		7	0		PD	0,093	**281**	1,0
**24**	N	neg.		8	0		NA	0,068	**285**	3,6
**25**	I	neg.		1	0		**NA**	**1,390**	**432**	**15,2**
**25**	N	neg.		2	0		PD	**2,810**	**671**	**31,7**
**25**	C	neg.		3	0		PD	**0,342**	**241**	1,2
**25**	C	neg.		4	0		PD	**0,157**	219	1,0
**25**	C	neg.		5	0		PD	0,098	**306**	0,6
**25**	C	neg.		6	0		PD	**0,352**	**315**	0,6
**25**	N	neg.		7	0		PD	**1,760**	**488**	3,4
**25**	N	neg.		8	0		PD*	**2,240**	**572**	**9,9**
**27**	I	BRAF V600E		1	0		NA	0,043	173	**8,1**
**27**	I	BRAF V600E		2	0		NA	0,079	**291**	2,1
**27**	I	BRAF V600E		3	0		**PD**	0,059	**373**	1,1
**27**	T	BRAF V600E		4	0		NA	0,049	226	0,8
**27**	T	BRAF V600E		5	0		NA	0,052	**273**	4,3
**27**	T	BRAF V600E		6	0		SD	**0,116**	**304**	1,1
**27**	T	BRAF V600E		7	0		SD	0,086	**242**	4,7
**29**	T	BRAF V600E	RAC1 P29L	1	11,35	8,06	NA	**0,320**	**278**	2,8
**29**	T	BRAF V600E	RAC1 P29L	2	0,04	0	NA	0,096	**323**	**11,3**
**29**	T	BRAF V600E	RAC1 P29L	3	0	0	**PD***	0,084	**519**	**6,1**
**30**	I	NRAS Q61R		1	0		NA	0,069	237	1,4
**30**	I	NRAS Q61R		2	0		SD	0,104	233	**9,4**
**30**	I	NRAS Q61R		3	0		SD	0,085	207	**10,1**

ID	Therapy	Mutation 1	Mutation 2	No.	Maf 1	Maf 2	Response	S100 (-0,105µg/l)	LDH (-240 U/L)	CRP (-5mg/L)
**30**	I	NRAS Q61R		4	0		SD	0,097	211	**5,2**
**30**	I	NRAS Q61R		5	0		SD	0,095	188	0,8
**30**	I	NRAS Q61R		6	0		**PD**	0,082	167	1,0
**30**	I	NRAS Q61R		7	0		PD	0,091	223	0,6
**30**	I	NRAS Q61R		8	0		PD	0,074	209	**7,8**

I	immunotherapy	*	new bone or brain metastases	LDH	lactate dehydrogenase
T	targeted therapy	CR	complete response	CRP	C-reactive protein
C	chemotherapy	PR	partial response	letters in bold	response to firstline therapy
N	no therapy	SD	stable disease	values in bold	increased parameters
No.	number of blood draws	PD	progressive disease		
MAF	mutant allele frequency	NA	not applicable		
