# Supplementary figures and images for: Circulating Tumor DNA as a Marker for Treatment Response in Metastatic Melanoma Patients Using Next-Generation Sequencing—A Prospective Feasibility Study
Source: Cancers (Basel). 2021 Jun 21;13(12):3101. doi: 10.3390/cancers13123101 (PMC8233754; doi:10.3390/cancers13123101)

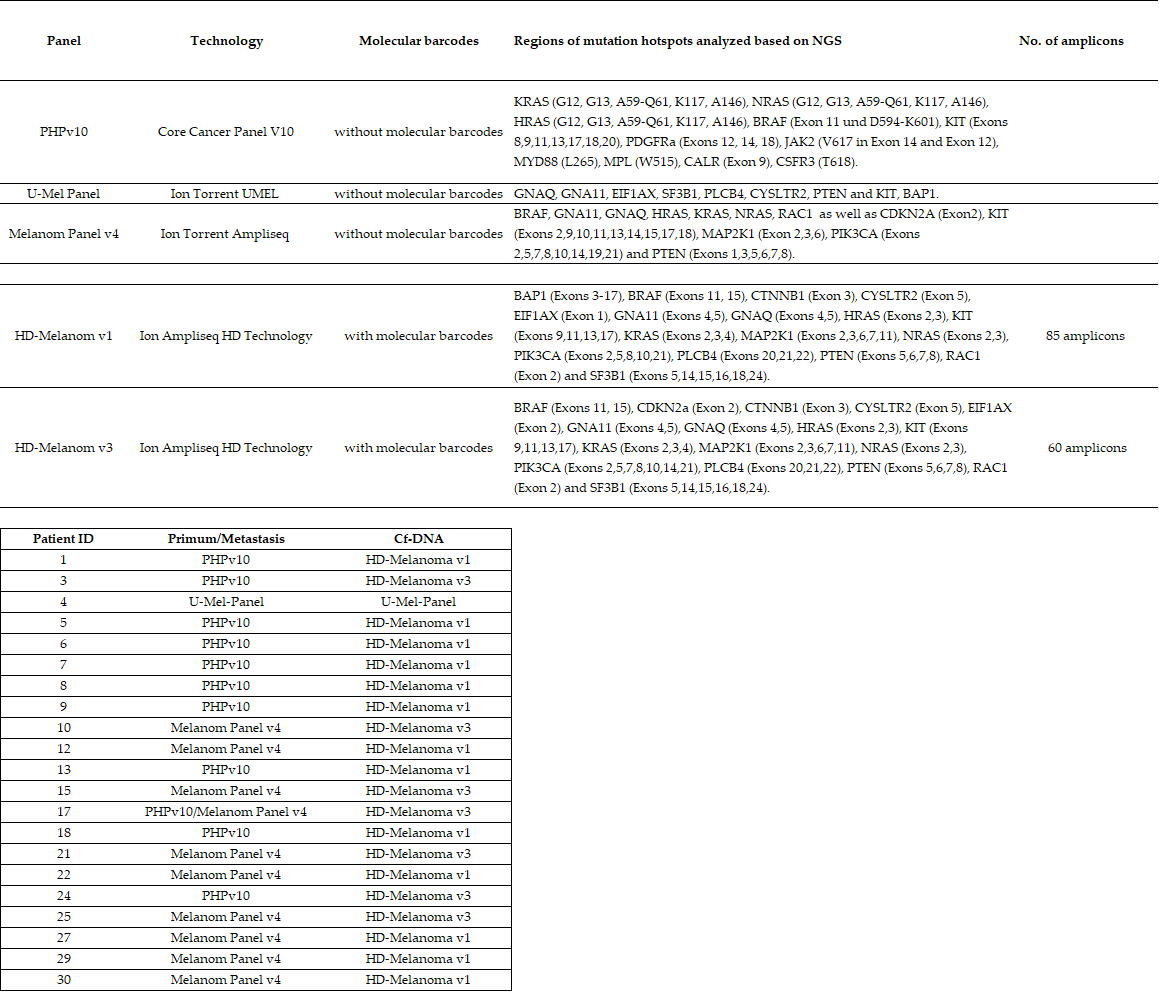

Supplement: Supplementary file 1 [file cancers-13-03101-s001.zip › Supplementary Table S1.tif]
